# T cell stimulation by dendritic cell-tumor cell hybrids is enhanced in the presence of free dendritic cells

**DOI:** 10.1186/2051-1426-1-S1-P232

**Published:** 2013-11-07

**Authors:** Mariana P Pinho, Patricia C Bergami-Santos, José Alexandre M Barbuto

**Affiliations:** 1 Department of Immunology, Institute of Biomedical Sciences, University of Sao Paulo, Sao Paulo, Brazil

## 

Dendritic cell-tumor cell hybrids (heterokaryons) are under investigation as a means for tumor immunotherapy since they should express MHC class I molecules from both cell types and, thus, present tumor antigens within an immunogenic context. The aim of this work was, therefore, to evaluate, in vitro, the immunostimulatory potential of these heterokaryons. To generate the hybrids, mature dendritic cells (mDCs) differentiated from peripheral blood of healthy donors were fused with cells from the breast cancer cell line MDA-MB-231 by applying an electric pulse. The hybrids expressed both the tumor antigen Her2 and the mDC marker CD11c (percentage of double-positive cells; Mix: 2.4 ± 0.5; Fusion: 13.2 ± 3.0; p<0.0001; n=8) and the molecules CD40, CD83, CD80 and CD86. They also expressed more MHC class I molecules at their surface (median fluorescence intensity; Mix: 17.8 ± 3.4; Fusion: 33.2 ± 7.2; p<0.05; n=4) which was proved to be due to the co-expression of molecules from both cell types. When co-cultured with autologous T lymphocytes for 5 days, the hybrids induced the same percentage of T cells positive for Foxp3 and T-bet but a higher percentage of GATA-3 positive T cells, when compared to the mix. Intriguingly, the hybrids induced lower T cell proliferation, when compared to the mix. However, when non-fused mDCs were added to the co-culture, T cell activation was significantly more intense than the sum of the activation induced by each cell type individually, as measured by T cell proliferation (percentage of cells with CFSE dilution; mDC: 9.7; Fusion: 6.3; mDC + Fusion: 47.8) and CD25 expression (percentage of positive cells; mDC: 9.9; Fusion: 9.7; mDC + Fusion: 57.4). Furthermore, addition of these free mDC to co-culture also induced more T-bet positive T cells. So, these data indicate that the induction/direction of immune responses by dendritic-tumor cell hybrids may be modified by the addition of non-fused DCs to the preparation, a strategy that may have clinical relevance.

## Financial support

CNPq, FAPESP (2009/54599-5 and 2012/10939-0).

